# Relationship between electroencephalographic data and comfort perception captured in a Virtual Reality design environment of an aircraft cabin

**DOI:** 10.1038/s41598-022-14747-0

**Published:** 2022-06-29

**Authors:** Giulia Ricci, Francesca De Crescenzio, Sandhya Santhosh, Elisa Magosso, Mauro Ursino

**Affiliations:** 1 Department of Electrical, Electronic, and Information Engineering “Guglielmo Marconi”, University of Bologna, Cesena, Italy; 2 Department of Industrial Engineering, University of Bologna, Forlì, Italy

**Keywords:** Neuroscience, Engineering

## Abstract

Successful aircraft cabin design depends on how the different stakeholders are involved since the first phases of product development. To predict passenger satisfaction prior to the manufacturing phase, human response was investigated in a Virtual Reality (VR) environment simulating a cabin aircraft. Subjective assessments of virtual designs have been collected via questionnaires, while the underlying neural mechanisms have been captured through electroencephalographic (EEG) data. In particular, we focused on the modulation of EEG alpha rhythm as a valuable marker of the brain’s internal state and investigated which changes in alpha power and connectivity can be related to a different visual comfort perception by comparing groups with higher and lower comfort rates. Results show that alpha-band power decreased in occipital regions during subjects’ immersion in the virtual cabin compared with the relaxation state, reflecting attention to the environment. Moreover, alpha-band power was modulated by comfort perception: lower comfort was associated with a lower alpha power compared to higher comfort. Further, alpha-band Granger connectivity shows top-down mechanisms in higher comfort participants, modulating attention and restoring partial relaxation. Present results contribute to understanding the role of alpha rhythm in visual comfort perception and demonstrate that VR and EEG represent promising tools to quantify human–environment interactions.

## Introduction

In an increasingly interconnected world, where people are constantly on the move, the development of new ways to improve passengers’ satisfaction is crucial for aircraft manufacturers and airline companies^1^. The passengers’ acceptance of a transportation system, and their willingness to use it again, are strictly related to the aircraft cabin features and spaces, which must elicit a sense of comfort and provide a pleasant flight experience^2^.

While traveling, the passenger is exposed to different stimuli related to thermal conditions, air quality, vibrations, posture, and visual stimuli coming from the environment that he/she experiences^3–5^. Each stimulus determines a comfort component that impacts the global comfort perception. Among such stimuli, the visual input significantly influences the passenger’s experience. Humans see an object's shape, size, glossiness, and lightness and form an impression of how comfortable it is. Furthermore, visual stimuli contribute to the formation of personal and peripersonal space perception, thus affecting the spatial perception of comfort, such as legroom, seat pitch, passage width, storage space^6,7^ as well as the sensation of having enough space to work, entertain or rest during the journey. Indeed, recent available research suggests that the visuomotor and visual perception of an environment could influence the experience within the environment itself^8,9^. In particular, according to Yao et al.^3^ while traveling, passengers often do not have an explicit cognitive task, and they have more spare time to visually explore the environment, analyzing its spatial and aesthetic features. Therefore, the visual experience is an essential construct of their overall comfort.

Understanding how spatial and aesthetic features of an aircraft cabin can affect the emotional state of users, as well as their perception of being in a comfortable environment, is of great relevance in promoting well-being and increasing passengers’ satisfaction^4^.

In this context, Virtual Reality (VR) represents a powerful tool to investigate subjects’ complex behaviors during their interaction with the external world. VR technology simulates environments in a realistic, immersive, and interactive way^5^, creating a sense of presence defined as the “sense of being in the virtual environment”. Indeed, through this technology we can represent real-life situations in highly controlled laboratory conditions^6^, thus allowing the simultaneous measurement of reliable behavioral and neurophysiological parameters^7,8^. There are different formats and devices for VR reproduction, which differ in their ability to induce a sense of immersion, i.e. the degree of isolation of the user from the physical world. For instance, immersive 3D virtual environments, compared to 2D, induce a greater subjective sense of presence, along with enhanced performances of spatial navigation, which correspond to a greater allocation of brain resources for cognitive and motor control^9^. Moreover, studies have shown that a highly immersive 3D virtual scenario generates realistic behaviors and physiological responses as if the subject is experiencing a real-life situation^10^. Another crucial factor that increases the sense of immersion is the capability to see the scenario from different perspectives and to move, allowing an embodied perception of 3D environments^11^. Indeed, through navigation around the environment, our visual system can learn about our surroundings^12^. For all these reasons Virtual Reality is an enabling technology for the involvement of end users since the early design phases of the product development process^13–16^.

Traditional methods for the evaluation of subjective satisfaction concern self-report questionnaires about their perceptions on the characteristics of the environment and their feeling of comfort and wellbeing^17^. Nevertheless, data obtained from questionnaires can lack accuracy as they are susceptible to subjective interference and external factors, preventing a true reflection of the emotional state of the user. Hence, the employment of this method alone is not sufficient to achieve a complete and objective understanding of how design features may condition the comfort perception.

Recent advances in psychophysical measurement techniques, in particular in wearable sensors technologies, make it now possible to achieve objective measurements of the subjects’ emotional and physiological states during their engagement with an environment, thus contributing to the improvement of the design process in order to create environments that meet human needs^18^. This integration of fields is known as neuroarchitecture and combines neuroscience and architecture to study the effects of built environments on the psycho-physiological states of its inhabitants^19,20^. In particular, through the electroencephalography (EEG) technique, one can measure brain activity non-invasively, obtaining neurophysiological data that are independent from the individual’s control^21–23^. Measuring brain activity represents a useful tool for emotion recognition^24^, allowing the investigation of neural correlates of various cognitive processes, such as comfort perception.

As it is well known, EEG activity exhibits oscillatory waves, located in different frequency bands. There is large consensus in the present neuroscience literature that this oscillatory brain activity is correlated with various cognitive phenomena, such as attention, memory formation, motor execution, and integration of information in large brain areas^25–28^. Among the others, alpha waves, typically located in the frequency band 8–12 Hz, have received much attention as a potential marker of a stress or arousal condition. Power in the alpha band is most prominent in the resting state^25^ and it is modulated by a variety of brain processes such as cognitive^29^, sensorimotor^30^ and psycho-emotional^31^. Depending on the type of stimulus or task demand, the alpha brain rhythm responds either with an Event-Related Desynchronization (ERD, alpha power decreases) or an Event-Related Synchronization (ERS, alpha power increases)^32^. Particularly, ERD occurs in the brain regions which are involved in task execution^33^, whereas ERS is observed in task-irrelevant regions, or those involved in the processing of potentially distracting stimuli^34^. Recent theories postulate that alpha-band oscillations act as a top-down inhibitory mechanism, implicated in attention selection^35^ and that a decrease in alpha power is related to an increased attention and, more generally, to a stressful condition^29,36^. Hence, changes in alpha power can represent a useful psychophysical marker able to discern between brain functional states characterized by a different arousal level^37^.

Finally, oscillatory phenomena in the brain are not localized in specific regions, but arise from the continuous exchange of information among various neural populations, which are implicated in the same task and synchronize their reciprocal activity. In this regard, the study of this “system of rhythms” can strongly benefit from the analysis of brain connectivity. The assessment of connectivity with modern processing techniques, such as Partial Directed Coherence^38^, Granger causality^39^ or Transfer entropy^40^ is becoming an important subject in modern neuroscience, to attain a deeper understanding of brain organizations during different tasks, and, more specifically, to assess the role of individual rhythms in cognition.

The aim of the present study was to assess the impact of aesthetic and spatial features of an aircraft cabin on the state of comfort/discomfort of users, by using a combination of VR instruments and EEG measurements. The idea is to combine subjective feedbacks on ergonomics, usability and perceived comfort, with neurophysiological responses, in order to provide an important framework for future design. Through the integration of a Virtual Reality (VR) platform with a wireless EEG system, it was possible to develop highly controlled real-size environments, in which a user could immerse and move, while simultaneously recording his/her brain activity^41,42^. The virtual scenario was reproduced using a CAVE system which provided a high degree of sensorial immersion^43^. At the end of the experimental session, subjects were asked to fill in a questionnaire on their perception of the cabin in terms of design spaces and aesthetics. Concerning the extraction of objective measures of brain activity, we focused on the alpha brain rhythm and evaluated both power density changes at different electrode locations, and connectivity among the electrode signals, using Granger causality.

Then, objective measures in the alpha-band, in terms of power and connectivity, have been compared with the subjective assessments extracted from the questionnaire. Specifically, we tested the hypothesis that comfort and discomfort sensations could be associated with different neural mechanisms and could activate alternative brain circuits. We are not aware of other studies that proposed this measure to investigate the perception of visual comfort/discomfort in an aircraft cabin.

The combination of these subjective and objective methods enables the study of designed aircraft cabins, and their impact on users’ experience, even before construction, providing a more accurate criterion for the design process. The study has been conducted within the framework of the European Horizon 2020 project CASTLE (Cabin Systems Design Toward Passenger Well-being), to foster an innovation process oriented to the optimization of the flying passenger’s experience using a Human Centered Design Approach^44^.

## Methods

### Participants

The experiments took place at the VLab of the University of Bologna, at the Department of Industrial Engineering (Forlì Campus). Thirty-one healthy volunteers (aged 18–33 years, mean ± std = 22.3 ± 3.5 years; 9 females) were recruited from university students. All participants were right-handed and had normal or corrected to normal vision and reported no medical or psychiatric illness. This study was approved by the local Bioethics Committee of the University of Bologna (file number: 187339, year: 2018) and performed in accordance with the relevant guidelines and regulations. Each participant signed an informed consent prior to the start of the experiment and all data were analysed and reported anonymously.

### VR environment and simulation scenario

Several experimental campaigns have been conducted along with the conceptual design phase of the CASTLE project, differing for updates in the design solutions or the Colour and Material Finishing features provided by designers. In the experimental campaign held in autumn 2019, for the purpose of this work, we maintained the experimental protocol and the virtual scenario already validated and described in detail in the De Crescenzio et al.^45^, integrated with EEG data acquisition. At this stage, the project is at a validation phase called CDR (Critical Design Review), when it is needed to exclude any major change in the components and any further refinement. Thus, the objective here is to rate a single VR configuration by possibly identifying levels of appreciation among a population.

The above-mentioned scenario is displayed on a CAVE Automatic Virtual Environment equipped with three, rear-projected, flat screens, for a total projection area of 7.5 × 1.5 m^2^. To allow the cabin environment to be navigated from a first-person perspective by a user moving on the CAVE floor, face and body tracking is implemented by capturing and filtering data provided by a Microsoft Kinect. For participants, the only encumbrance generated by the VR set-up are simple 3D stereoscopic glasses to create the impression of depth on the screens. The different 3D models populating the environment are representative of the cabin items of interest such as fuselage, seat, lavatory, galley, cabin lining, Flight Attendant Seat and stowage bins. An avatar is displayed in the main egocentric view of the scene. Moreover, in the lower bottom corner of the right screen, an exocentric view of the avatar is reproduced in order to support the user’s proprioception.

### Experimental protocol

Each participant underwent a single experimental session. During the entire session, the participants wore a wireless EEG device and they were asked not to speak and to limit their movements, with the exception of one phase (the *interaction* phase) where they were explicitly asked to move and interact with the virtual cabin.

In order to avoid to distract the participants and enhance their sensory immersion, the lights of VR laboratory were kept off and operators stayed in the background throughout the entire session.

Before the start of the main experimental session, the baseline (*base*) EEG signal was acquired for each participant. It consisted of a 5-min resting-state with eyes open, during which the participants seated centrally to the CAVE at approximately 2 m away from the screens, in absence of any stimulation, either visual or auditory, as VR screens were kept off and no sound was played. This phase was explicitly designed to elicit a baseline relaxation state in each participant.

At the end of baseline phase, the participants, after wearing the stereoscopic 3D glasses, were asked to stand on the CAVE platform and were briefly trained to navigate the virtual environment, in order to familiarize with the avatar and with the interactive features of the virtual cabin (auditory and visual feedbacks of collision effect). It should be noted that the virtual scenario used during this training phase (a visual stowage on a black background) was different from those of the main experimental session.

The subsequent main experimental session was structured into four phases, overall lasting 18 min.

The first phase, named *r1*, consisted in a 5-min eyes-open resting state without VR stimulation. As in the baseline acquisition, the participants were seated centrally to the CAVE, with the difference that a background sound, simulating an aircraft in motion, was turned on and was kept on for the rest of the experimental session. The second 5-min phase, named *r1VR*, consisted in a first static VR immersion phase. Here, the VR screens were switched on and were kept on until the end of the session. During this phase, the participants remained seated while visually exploring the static VR environment in which they were immersed, trying to capture both spatial and aesthetic aspects.

**Figure 1:**
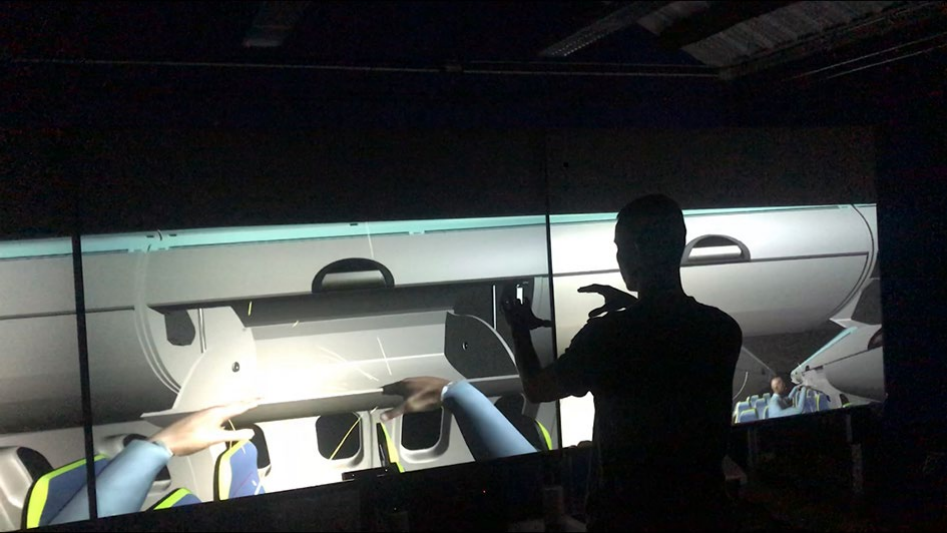
Interaction of the subject with the virtual cabin.

Specifically, the presented scenario consisted in an immersive view of a Regional Cabin Aircraft including a part of the aisle, the seats and the stowage bins. The third 3-min phase consisted in the *interaction* of the participants with the virtual cabin. Here, the participants stood up and moved in order to explore spaces and interact with the objects of the environment (Fig. 1). To this end, the chair was temporarily removed from the CAVE to allow a better navigation. Finally, the last 5-min phase, named *r2VR*, consisted in a second static VR immersion phase. During this phase, the experimental conditions were the same as in the first VR immersion phase (*r1VR*), with the participants seating in front of the screens and in the same static scenario shown previously.

At the end of the experiment, each participant filled in a questionnaire (see section “Questionnaire”) for subjective rating of comfort induced by the aesthetic and spatial features of the VR cabin shown during the main experimental session.

The EEG signals acquired in this experiment were used to investigate whether alpha-band EEG features (power and connectivity) during VR immersion differed depending on the subjective level of comfort (low vs high) elicited by the VR environment, as emerged from the questionnaire. It is worth noticing that the interaction phase (int) was excluded from the analyses. Indeed, in this phase, EEG signals were strongly corrupted by movement artifacts that couldn’t be corrected in a reliable way. Moreover, during this phase additional motor mechanisms were involved, influencing brain rhythms and likely confounding the effects related to comfort sensation. This does not mean that the effects of interaction phase were neglected: indeed, during the post-interaction phase (r2VR), comfort and appreciation of the VR environment, and related EEG features, were likely affected by the outcome of this interaction.

### Questionnaire

The questionnaire consisted of 9 questions in total (Table 1). In the first 8 questions participants were asked to evaluate the spatial and aesthetic properties of some cabin features, such as seats, cabin lining and stowage and they answered the question “Please, express how much you agree with each statement” using a 5-point Likert scale, where 1 = strongly disagree, 2 = mostly disagree, 3 = slightly agree, 4 = mostly agree, 5 = strongly agree. The last question ('How much did you like the cabin?') aimed to capture the participant's overall appreciation of the cabin. Since the question was more general, a broader 10-point scale from 1 (minimum) to 10 (maximum) was used to allow subjects to define their level of appreciation more precisely.

**Table 1:** Overall cabin scenario questionnaire.

1. The seat seemed easy to access	Seat
2. The seat appeared to have enough space to stretch my legs	
3. I had the feeling of being in a spacious environment	
4. The space to access the seat and the leg room appeared sufficient	
5. I was pleased with the style/aesthetics of the cabin lining	Cabin lining
6. I was pleased with the style/aesthetics of the stowage bins	Stowage bin
7. The stowage bin appeared spacious enough to easily load my luggage	
8. The stowage bin seemed easy to reach/use	
9. How much did you like the cabin?	Overall cabin liking (10 points)

### EEG acquisition and pre-processing

For each participant, fourteen electrodes (F3, F4, C3, C4, T7, T8, Pz, PO3, PO4, PO7, POz, PO8, O1, and O2) were collected at 125 Hz sampling rate and 24-bit using a wireless EEG device (OpenBCI, https://openbci.com/) and pre-processed offline using MATLAB (MathWorks Inc., Natick MA, USA). Pre-processing included: filtering (0.75–60 Hz band-pass filter plus 50 Hz notch filter); removal of the *interaction* phase; artifact removal via Independent Component Analysis (ICA); extraction of the 5-min EEG data segments corresponding to the single phases *base*, *r1*, *r1VR*, *r2VR;* Individual Alpha Window (IAW) identification^46^ using the *base* signals.

During these stages, two subjects were excluded for further analyses (one because of a severe ECG artifact, not removed by ICA; the other because of extremely high variability across experimental phases that risked to bias the entire sample trend).

### Alpha band analysis: power and connectivity

For each participant, and each 5-min phase (*base, r1, r1VR, r2VR*), this analysis involved: (i) alpha power computation over the IAW for each electrode (*channel-wise alpha power*); (ii) averaging of the channel-wise alpha power over the fronto-central-temporal (FCT) electrodes and parieto-occipital (OCC) electrodes obtaining *regional-wise alpha power*; (iii) normalization of both channel-wise and regional-wise alpha powers to the corresponding values in the base phase; (iv) computation of Granger Causality (GC) spectrum (function of frequency) between all pairs of electrodes and in both directions; (v) computation of the mean value of each GC spectrum in the IAW, obtaining alpha-band GC, and normalization to the corresponding value in the base phase.

### Total subjective score and dichotomous subdivision

Analysis on subjective data was carried out considering the scores of the questionnaire that each participant filled at the end of the experimental session. Hence, the nine scores (one for each question) were summed up for each participant, obtaining a *Total Subjective Score* (TSS). TSS ranged from a minimum value of 9, as the minimum assignable value to each question was 1, to a maximum value of 50, as the maximum assignable value to eight out of nine questions was 5 and only for the last question was 10. In order to investigate differences in neural mechanisms underlying a different level of VR-induced comfort, subjects were divided in two classes by considering the median TSS value (*md*TSS = 35): group G1 consists of participants (16) with a TSS less than or equal the median, i.e. who perceived lower comfort in the virtual cabin; group G2 consists of participants (13) with a TSS above the median value, i.e., who showed a greater appreciation of the virtual cabin.

### Statistical comparison

The analysis of alpha power is presented in two steps.

The first step considered the entire set of participants (without group subdivision) and provided an overall assessment of how sensory stimulation in the different experiment phases affected alpha brain rhythm. For this purpose, we statistically compared the normalized alpha power in each of the three experimental conditions of background acoustic stimulation (*r1*), pre-interaction VR-immersion (*r1VR*) and post-interaction VR-immersion (*r2VR*) with the baseline condition (base), separately for each macro-scalp region (FCT and OCC). A two-tailed permutation-based *t* test for dependent samples was used for each comparison and Bonferroni correction was applied (separately for each scalp macro-region).

The second step investigated whether different levels of comfort (lower and higher) elicited by the VR cabin were associated with differences in alpha-band EEG features (power and connectivity) during VR immersion. Since r1 did not involve VR immersion, these analyses were performed on phases r1VR and r2VR, by statistically evaluating differences between the two groups of participants (G1 and G2) in each phase. The normalised alpha power, both *regional-wise* and *channel-wise,* was compared by using the one-tailed permutation-based *t* test for independent sample, under the hypothesis that subjects who particularly enjoyed the cabin (G2) also felt more relaxed during its visual exploration, showing greater alpha-band power. For each phase separately, multiple comparison correction was based on Bonferroni in case of regional-wise analysis and on maximum t-statistic in case of channel-wise analysis. The statistical comparison of the normalised alpha-band GC between the two groups was performed using the one-tailed non-parametric permutation test for independent samples. In this case, uncorrected p-values were considered, due to the high number of involved variables, i.e. 14 × 13 connections, making correction requirement highly demanding; however, different levels of significance were distinguished (see section “VR immersion effects on normalised alpha power: behaviour of group G1 and group G2”). A more detailed version of the Methods can be found in the Supplementary Information

## Results

### Questionnaire results

No specific issue arose from the virtual navigation of the cabin since all subjects were able to complete the navigation and interact with the seat and stowage bin.

**Figure 2:**
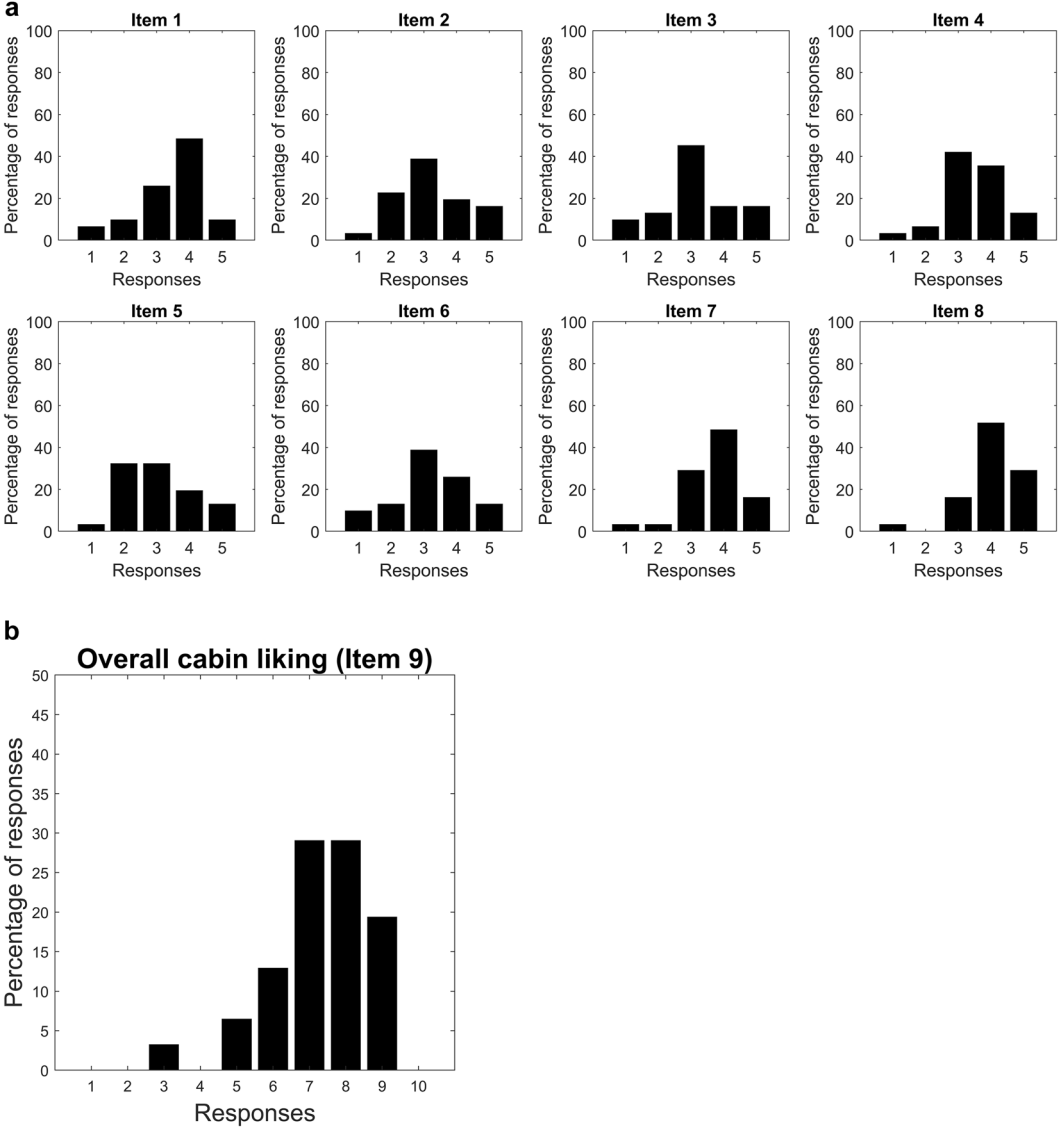
Top panel: histograms showing distribution of responses to the Items 1–8. Bottom panel: histogram showing distribution of responses to the Items 9 (‘Overall Cabin linking’).

The top panel of Fig. 2 shows the responses distribution of the 31 subjects to the questions about the seat (Items 1–4), the cabin lining (Items 5) and the stowage bin (Items 6–8). The histograms show the liking trend of these cabin features among subjects, considering their spatial and aesthetic characteristics.

Concerning the seat (Items 1–4), most of the participants (Item 1: ≅ 83%, Item 2: ≅ 74%, Item 3: ≅ 77%, Item 4: ≅ 90%) rated the items with a score of 3 or above, showing a general appreciation of this aspect of the cabin. Additionally, about half of the participants (≅ 45%) only slightly agree (voted 3) that they had the feeling of being in a spacious environment (Item 3). About 35% of participants mostly disagreed (voted 1 or 2) about being pleased with the style/aesthetics of the cabin lining (Item 5). Finally, regarding the stowage bin (Items 6–8), most of participants (Item 6: ≅ 77%, Item 7: ≅ 93%, Item 8: ≅ 97%) mostly agreed (scored 3 or more) about being pleased with its style and design and about it being easy to operate.

The bottom panel of Fig. 2 shows the distribution of responses to Item 9. The cabin was overall rated positively by participants, that responded 5 or above (on a scale of 1 to 10), except for a single subject who gave a score of 3. Indeed, more than 77% of the sample responded 7 or above, suggesting that the way the cabin is constructed was appreciated by participants.

**Figure 3:**
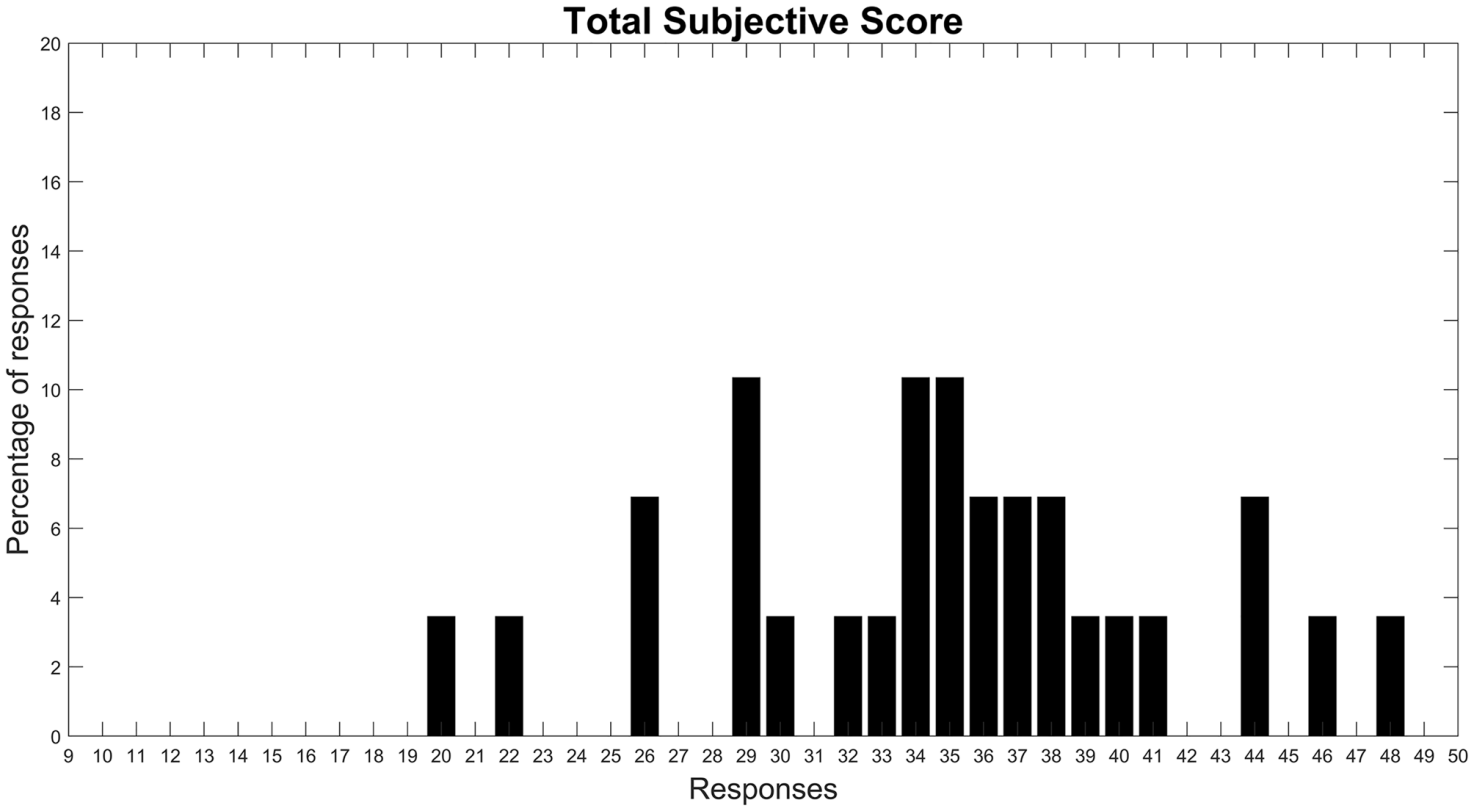
Histogram displaying the Total Subjective Score distribution (from TSSmin = 9 to TSSmax = 50).

To compare subjective and objective data (alpha-band EEG features), all the above-mentioned aspects (Items 1–9) were summarized in the Total Subjective Score index. Figure 3 shows the distribution of the TSS index among 29 participants. It should be noted that, in order to compare TSS with objective data, the two subjects who were excluded during EEG signal processing were also removed for the calculation of this index. The TSS distribution shows that the aesthetic and spatial features of the cabin were generally appreciated across participants. Indeed, less than 25% of subjects gave an overall rating below the middle scale point (around $TSS = 29$). With the aim of dividing the subjects into two distinct groups, only those subjects who particularly enjoyed the cabin, i.e. those whose TSS index resulted strictly above the median value (35), were included in the high comfort group (G2).

### Sensory immersion effects on normalised alpha power: overall assessment across all participants

**Figure 4:**
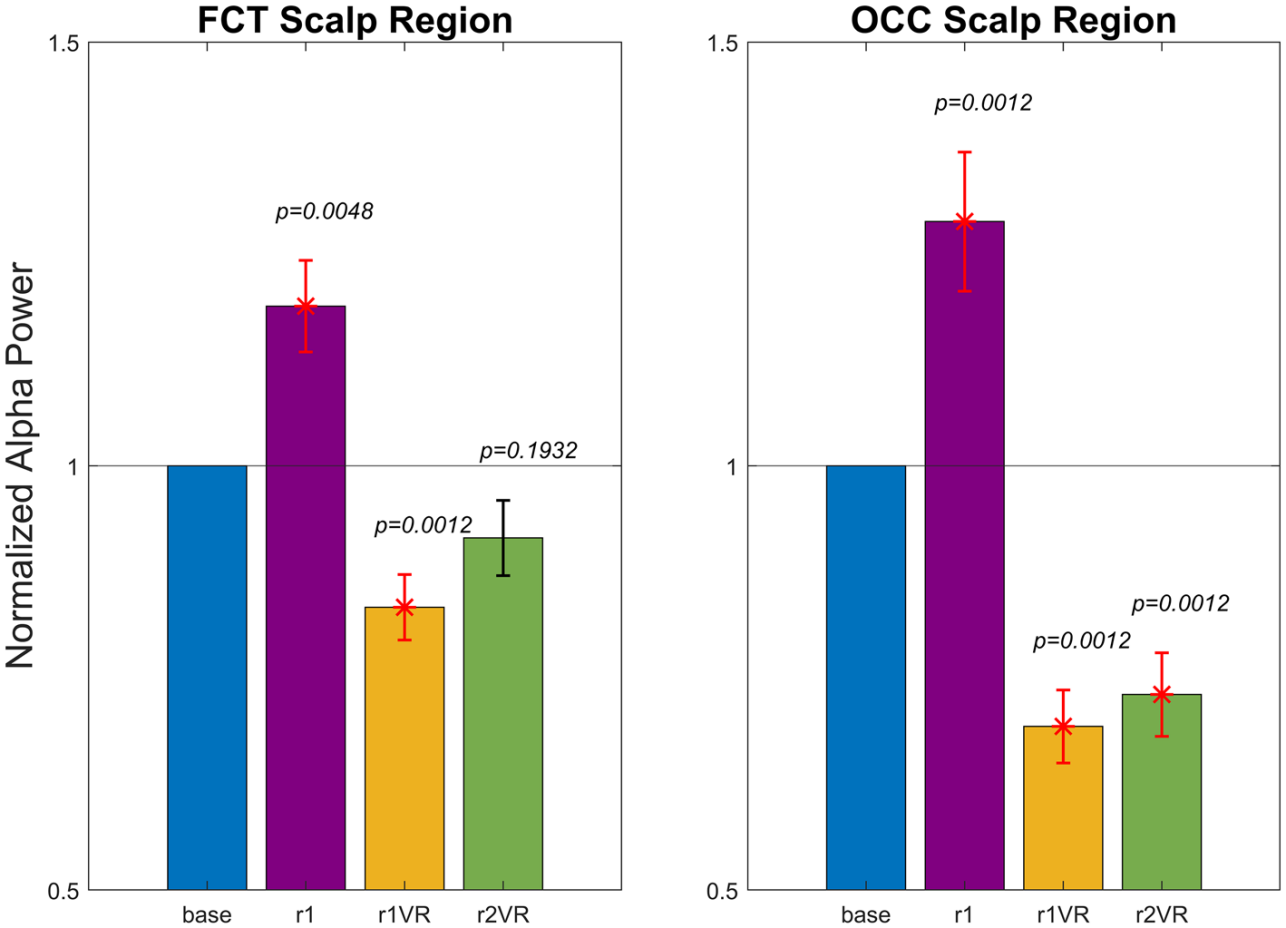
Normalized alpha power, averaged across all participants (mean ± sem), over the fronto-central-temporal FCT scalp region (left panel) and over the parietal-occipital OCC scalp region (right panel) during each of the three phases (r1, r1VR, and r2VR) of the sessions. Since normalization was performed relative to baseline condition (see “Methods” section), normalized alpha power was equal to 1 in the base phase in each participant and region, and was reported for clarity. The figure also shows results of the two-tailed permutation test for dependent samples obtained comparing the normalized alpha powers in each phase r1, r1VR and r2VR with the *base*, separately within each region. Error bars are coloured in red when the considered phase compared to the *base* was statistically significant (Bonferroni corrected p-value < 0.05), while these are coloured in black in the absence of significance.

Figure 4 displays the bar plots (mean ± sem) obtained across 29 participants and shows the change of normalized alpha power during the phases *r1, r1VR*, and *r2VR* compared to baseline, in each of the two scalp macro-regions, the fronto-central-temporal (FCT: left panel) and the parieto-occipital (OCC: right panel).

The *r1* phase, during which only the background noise was played without any visual stimulus (screens OFF), was characterized by a large alpha power increase, more evident in the OCC region, possibly denoting that such background noise could have enhanced the relaxation state of participants prior to VR-immersion phases (*r1VR* and *r2VR*). At variance with *r1*, both these two phases were characterized by a relevant reduction in alpha power, induced by the immersion in the VR scenario. This reduction was more dramatic in the OCC region. Moreover, alpha power reduction was more pronounced in the pre-interaction phase *r1VR*; in *r2VR,* after the interaction with the virtual cabin, alpha power exhibited a less decline especially in FCT regions. Finally, a crucial aspect emerges by comparing FCT and OCC scalp regions. Indeed, although alpha power pattern was similar in both macro regions, alpha power in FCT region was always less affected by the external stimuli (acoustic and visual) than the OCC region.

Statistical comparisons confirmed the following results: normalised alpha power in *r1* and *r1VR* departed significantly from the baseline condition in both FCT and OCC regions, while in *r2VR* decreased significantly only in the parieto-occipital region (OCC) and not in the fronto-central-temporal region (FCT).

### VR immersion effects on normalised alpha power: behaviour of group G1 and group G2

This section presents the *regional-wise* and *channel-wise* analysis of the normalised alpha power in VR immersion phases, *r1VR* (pre-interaction) and *r2VR* (post-interaction), obtained after splitting the entire set of participants in two groups (G1 and G2) considering their perception of comfort in the virtual cabin (lower and higher comfort, respectively).

**Figure 5:**
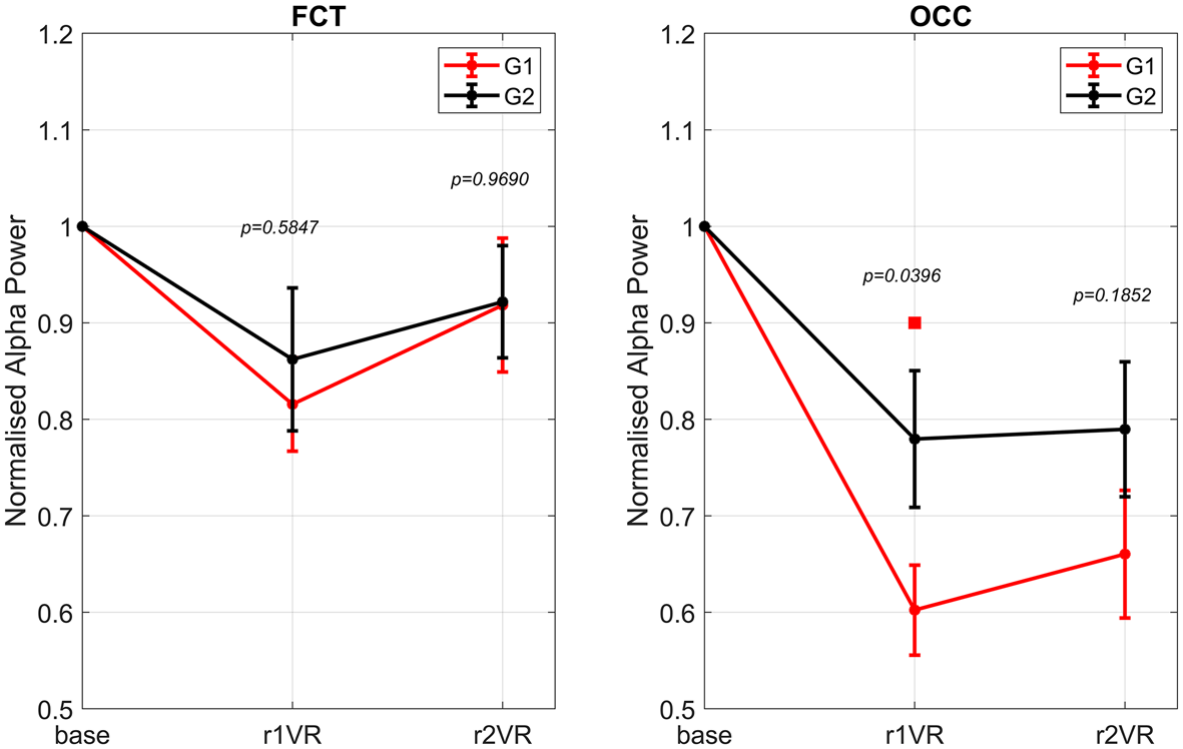
Regional-wise analysis in group G1 and G2: normalized alpha power during each of the three phases (*base*, *r1VR*, and *r2VR*), averaged across participants of the same group (mean ± sem), over the two scalp macro-regions (fronto-central-temporal FCT (left panel); parieto-occipital OCC (right panel)). Red square denotes statistical significance difference between the two groups (one-tailed permutation-based *t *test, p-value < 0.05 Bonferroni-corrected for multiple comparisons). The only significant difference between groups G1 and G2 is achieved in r1VR phase and over the OCC scalp region (corrected p-value = 0.0398).

In the *regional-wise* analysis, G1 and G2 behaviour during VR immersion (r1VR and r2VR) was studied in the two macro-regions (FCT and OCC), as previously done across all participants in Fig. 4. For each scalp region, the mean ± sem values (across participants of the same group) of normalized alpha power during VR immersion phases are depicted in Fig. 5 (FCT: left panel; OCC: right panel).

In both groups, the reduction in alpha power was larger in the OCC region compared to the FCT region, confirming the higher influence of VR-stimulation on the parieto-occipital alpha rhythm. Alpha power decrease observed in FCT regions was quite similar in G1 and G2 and in both VR-stimulation phases. Conversely, a more pronounced group difference occurred in the OCC region. In particular, statistical results show that group G1 was significantly different from group G2 only in the phase *r1VR* (before the interaction with the cabin) and over the parieto-occipital scalp region.

**Figure 6:**
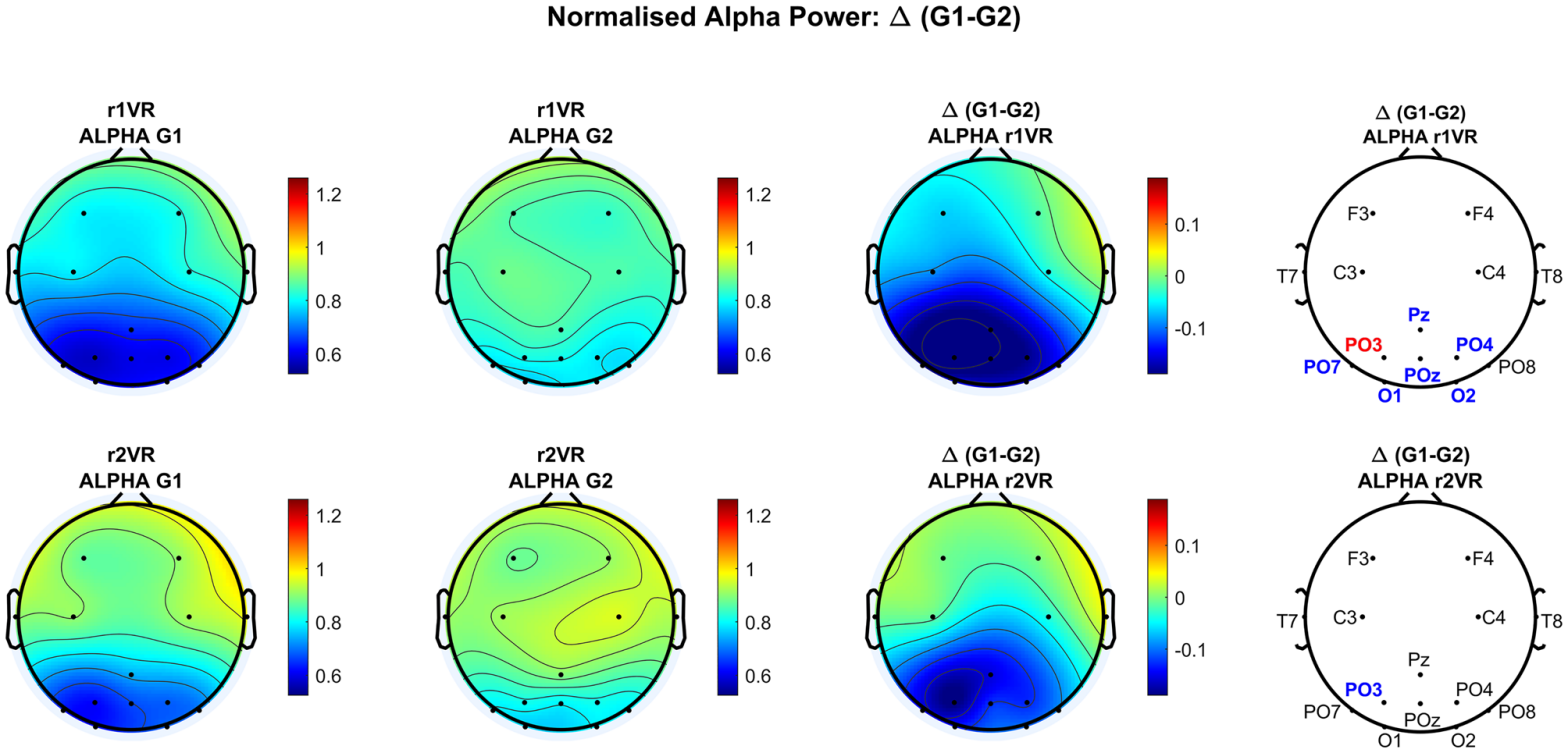
Channel-wise analysis in group G1 and G2: scalp maps and statistical analysis for normalised alpha power. Each row refers to a VR phase: *r1VR* on the first row and *r2VR* on the second row. The maps in the first and second column represent the normalised alpha power averaged across participants of group G1 and G2 respectively. The maps in the third column represent the normalised alpha power difference between G1 and G2. In the cartoon heads of the fourth column, the results of the statistical comparison between the two groups (one-tailed permutation-based *t* test for independent samples) are shown: the blue markers denote the uncorrected statistically significant electrodes (uncorrected p-value < 0.05) while the red markers denote electrodes where significance survived the correction for multiple comparisons (maximal statistics, corrected p-value < 0.05). The only significant difference between groups G1 and G2 after p-values correction was achieved in *r1VR* phase and for the PO3 electrode (corrected p-value = 0.0482).

Figure 6 shows, arranged in rows, the outcomes of the *channel-wise* analysis obtained in *r1VR* (first row) and *r2VR* (second row). The first and the second column of the figure represent the scalp maps of normalised alpha power of group G1 and G2 respectively, the third column shows the scalp map differences between G1 and G2, and the fourth column shows the statistically significant channels resulted from the comparison between groups (red and blue denote electrodes statistically significant with and without correction for multiple comparisons, respectively).

In the first VR phase (*r1VR*) a more dramatic reduction of alpha power occurred in group G1 compared to group G2. Furthermore, as confirmed by statistical analysis results, this power reduction was more pronounced in the parieto-occipital electrodes, with a slight tendency towards the left hemisphere. A similar effect arises in the post-interaction phase *r2VR*, although less significant.

### Effect of the VR immersion on spectral Granger causality in the alpha band: behaviour of group G1 and group G2

**Figure 7:**
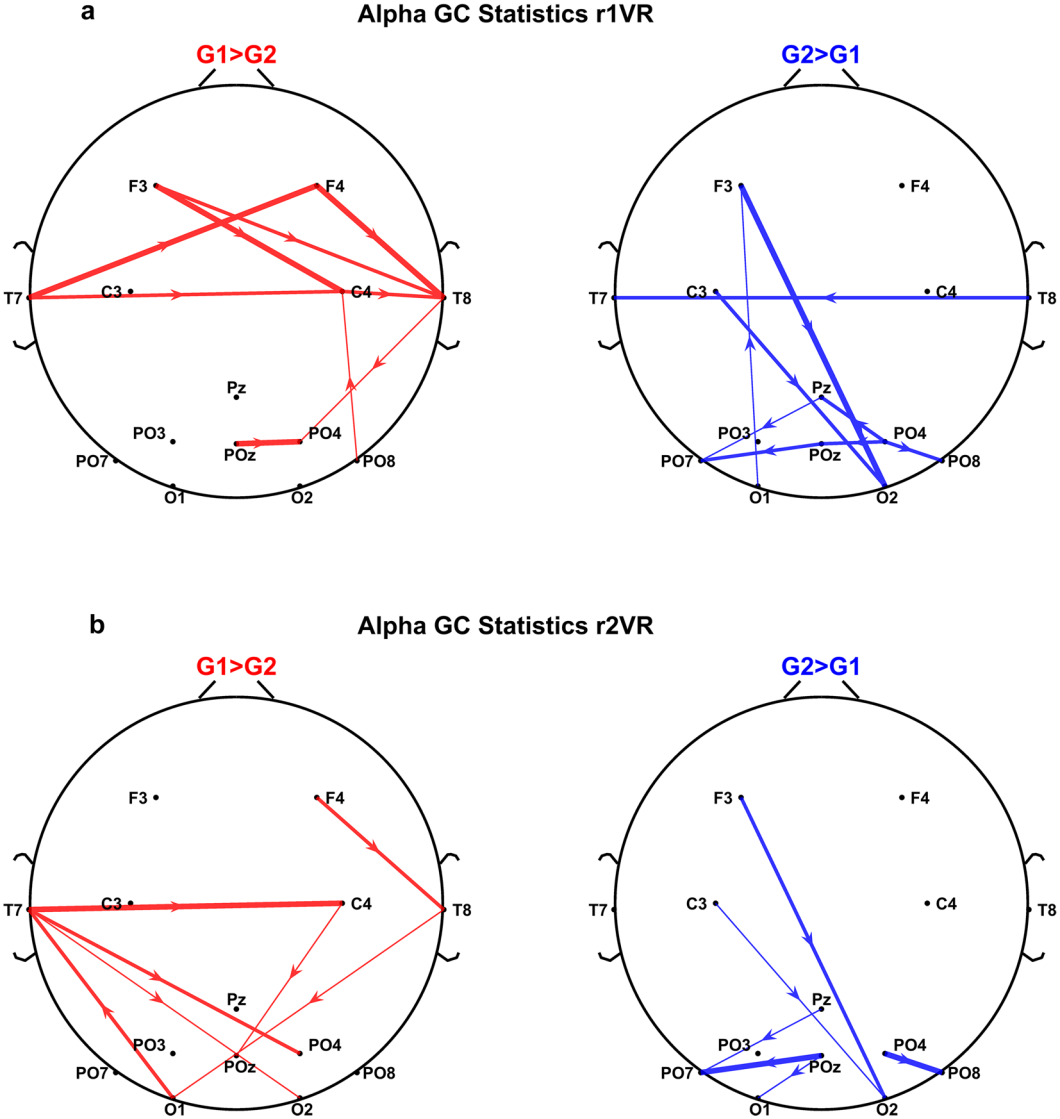
Results of the spectral Granger Causality in the alpha band between scalp electrodes. The cartoon head shows the connections that are significantly different between group G1 and group G2: in particular, red lines refer to normalized connections higher in group G1, whereas blue lines denote normalized connections higher in G2, in each VR-immersion phase (r1VR: top panel, r2VR_bottom panel). The displayed arrows correspond to significant connectivity changes at p-value < 0.05 (uncorrected one-tailed permutation-based *t* test) and the thickness of the line denotes three levels of significance, i.e., thinnest line: $0.025 \le p < 0.05$; middle line: $0.01 \le p < 0.025$; thickest line: $p < 0.01$.

Figure 7 shows the outcomes of the statistical analysis in *r1VR* (top panel) and *r2VR* (bottom panel) concerning the normalised Granger causality in the alpha-band. Only connections showing significant differences between the two groups were plotted (uncorrected p-value < 0.05).

For each phase, the figure shows in red the electrode connections that are stronger in G1 (lower perceived comfort), while in blue the electrode connections that are stronger in G2 (higher perceived comfort).

In *r1VR* phase (top panel), the group G1 (in red) exhibits a higher alpha-band connectivity in the anterior region of the scalp: the emerging circuit involved an information flow within the FCT region and with a tendency from left to right. Besides this network, only a few connections involving parieto-occipital electrodes emerged.

Instead, in the same VR-immersion phase (*r1VR*) the alpha-band network in G2 (in blue) exhibits a stronger posterior OCC network linking parieto-occipital electrodes. This result may reflect the lower reduction of alpha power in the parieto-occipital region for G2, as shown in the previous sections. In addition, a strong top-down mechanism occurs in G2 between left fronto-central electrodes and occipital electrodes. Besides the two main mechanisms described above, only few connections appear in G2 (one weak bottom-up and one temporal inter-hemispheric connection).

However, in *r2VR* phase, after the subject's interaction with the virtual cabin, the differences in G1 group connectivity vs. G2 drastically change. In fact, in r2VR, the alpha-band connections of the G1 group, which in r1VR were localised in the FCT macro-region, move towards the parieto-occipital electrodes and show an increased top-down regulation. This new G1 network may reflect the slight increase in alpha power observed in *r2VR* immersion phase, after the interaction with the cabin. Conversely, the G2 network in *r2VR* retains the same mechanisms observed in *r1VR*, confirming a greater top-down information flow and the parieto-occipital circuit.

## Discussion

In this study, we evaluated whether EEG alpha power changes can be employed to discriminate the *visual* comfort experienced by subjects in a virtual aircraft cabin and to investigate the related connectivity differences. This represents a fundamental stage in the design process of a cabin environment. Indeed, although other factors influence the emotional state of a passenger, the effect of visual stimulation is undoubtedly one of the most relevant when assessing comfort in an environment^47,48^. Furthermore, the combination of different types of stimulation (visual, auditory, proprioceptive, etc.) would lead to other concurrent effects on brain rhythms in several highly distributed cortical regions, making it challenging to attribute EEG alterations to a specific sensory dimension. From the perspective of product development, we believe that a step-by-step procedure, starting with visual factors and progressively adding other aspects, would allow better investigation of the different ‘comfort dimensions’ and the application of more specific project modifications.

In the present work, we focused attention on the alpha rhythm, which is the dominant rhythm in awakeness and is believed to play a relevant role in establishing the brain’s internal state^49^. Recent studies suggest that the alpha rhythm is related to vigilance^37^, awareness^50^, processing of spatial features^51^, and attention^52,53^. Furthermore, Schubring et al. observed that brain oscillations in the alpha/beta-band might be a valuable marker of emotional stimulus processing^54,55^. Although alpha brain rhythm modulation (ERD/ERS) has been reported in various cognitive domains, its role is primarily associated with the processing of visual stimuli^27,32^.

The choice to explore both alpha power and connectivity was dictated by the observation that alpha rhythm can have different cognitive functions and involve two complementary mechanisms: while a decrease in the amplitude of alpha oscillations (ERD) is strongly associated with an increase in cortical excitation, an alpha synchronization among different regions can be related to top-down regulation^56,57^. Analysis has been conducted at the EEG scalp level since the limited number of electrodes precludes a reliable reconstruction of cortical sources.

At first, the EEG data analysis was performed on the entire data sample (all subjects) to grasp the general trend of alpha-band power in the different phases of the experiment (*r1*, *r1VR*, *r2VR*) and both in fronto-central-temporal (FCT) and parieto-occipital (OCC) macro-regions of the scalp.

Our results confirm that during the immersion in the virtual aircraft cabin (*r1VR* and *r2VR*), when subjects’ attention is devoted to the environment, the alpha power significantly decreases (ERD) compared to baseline, and power reduction is more marked in the OCC region.

This finding agrees with previous neurocognitive works on alpha rhythm modulation, which demonstrated that an increase in visual attention reflects a decrease in alpha power (ERD) in parieto-occipital areas^36,58–60^. Further studies have also shown that in these areas prestimulus alpha power is inversely related to perceptual sensitivity and awareness^49,50,61^. Moreover, recent findings demonstrated that posterior alpha power decreases more in response to a conscious visuospatial stimulus^62^ or attended task-relevant signals^63^ compared to an unseen stimulus, as well as during the processing of emotional pictures^54,55^.

Although less relevant to the purpose of the study, another result emerged concerning the *r1* resting phase, where only the sound simulating the internal noise of an aircraft was reproduced while the screens were turned off. In this case, alpha power significantly increases with respect to the baseline in both FCT and OCC scalp regions, suggesting that such sound may have induced a relaxation state in participants before the VR-stimulation phases (*r1VR* and *r2VR*). Indeed, if a pronounced reduction in alpha rhythm (ERD) reflects cortical excitation, a dramatic increase of this rhythm (ERS) can usually be observed during the inhibition of task-irrelevant brain areas or during relaxed wakefulness without higher cognitive load (cortical idling)^34^.

The second analysis step was conducted after splitting subjects into two groups (G1: low comfort, G2: high comfort) to investigate the modulation of alpha power as a possible index of comfort perception in the virtual aircraft cabin. For this purpose, subjects were subdivided according to the 'Total Subjective Score,’ which summarizes their satisfaction with the cabin in terms of spaces, aesthetics, and overall liking.

Results on alpha power in the two VR-stimulation phases (*r1VR*, *r2VR*) demonstrate that group G1, whose subjects experienced a lower state of comfort in the cabin, show a greater ERD in the OCC region compared to group G2 whose ERD is milder, although present.

Of course, it is not easy to summarize the exact reasons for these differences, but some basic assumptions can be made. Subjects who experienced lower comfort could be characterized by higher awareness, be more stressed, or pose more attention to some features of the cabin. Conversely, the other class of subjects may have countered the intense visual stimulation imposed by VR through the higher feeling of comfort (or relaxation) elicited by the cabin design. In particular, as the largest inter-group differences are confined to OCC regions, we can hypothesize that occipital alpha rhythm is simultaneously conditioned by both visual stimulation and subjects’ psychophysiological states of comfort in the cabin environment.

Furthermore, another phenomenon emerges from the study. Before any subjects’ interaction or movement within the cabin (*r1VR*), the alpha-band power is greatly affected by the immersive environment, showing a more evident ERD. In this condition, alpha ERD appears significantly correlated with the perceived comfort. Conversely, alpha ERD becomes less pronounced in *r2VR,* especially in the FCT regions, where differences between G1 and G2 become negligible. Since the fronto-central-temporal brain areas are involved in the motor network, we can hypothesize that ERD mitigation in *r2VR* is driven by sensorimotor activity, which modulates the alpha-band ERD/ERS after movement^34,64,65^. These results hint that navigation in the space can reduce the discrimination ability of alpha ERD as a possible measure of visual comfort. However, as the main group differences in our study emerged in phase *r1VR* (pre-movement) and in the OCC brain region (the one mainly affected by visual processing), we think this aspect does not jeopardize our main results.

Hence, the obtained findings suggest that, in perspective, the present methodology can be exploited, together with other psychophysical measurements (such as heart variability or skin conductance) to quantify the visual appreciation of aircraft design and, more generally, to evaluate arousal, emotion, or stress conditions in a VR environment.

Several studies recently analyzed EEG in VR, emphasizing the role of brain rhythms^66,67^. However, most of these studies were focused on motion responses. Among the others, Vecchiato et al.^68^ measured the EEG correlates of architectural perception in the theta, alpha, and mu bands and in cerebral circuits of sensorimotor integration, spatial navigation, and embodiment. Ehinger et al.^69^, through a VR system in which the subject was traversing a triangular path, observed an alpha suppression in parietal, occipital, and temporal clusters during turning movement, ascribing it to kinesthetic and vestibular information. Hofmann et al.^70^ established the association between emotional arousal and parieto-occipital alpha power during an immersive VR experience, confirming the value of this tool for studying human emotions in circumstances similar to everyday life.

The present study goes in the same direction, supporting the idea that alpha power changes can be exploited as a neural marker of perception and comfort, more generally of emotional states, in artificial landscape design, such as aircraft cabins.

While changes in occipital alpha power are inversely related to awareness, the synchronization of alpha rhythm between brain regions likely reflects the presence of top-down control mechanisms^71^. Different functions have been ascribed to this control. One hypothesis is that alpha–band connectivity from the frontal to the occipital cortex may transmit information about internally generated predictions, thus introducing a kind of prior information that biases external perception^56,72^. A second hypothesized mechanism involves attention: a network of fronto-parietal cortical regions [the so-called dorsal and ventral attention networks, which include the frontal eye fields, the intraparietal sulcus, the ventral frontal cortex, and the temporoparietal junction, see Vossel et al.^71^ mainly working in the alpha band, has been proposed to be responsible for a top-down attention modulation to guide sensory perception^51,73,74^. D’Andrea et al.^53^ observed that alpha-band phase-synchronization is modulated by attention according to a parieto-occipital top-down mechanism and that the right putative Frontal-Eye-Field, not the left, is recruited in the deployment of spatial attention. Lobier et al.^75^ found that visuospatial attention is associated with robust and sustained long-range synchronization of cortical oscillations in the high-alpha band, connecting frontal, parietal, and visual regions. Benedek et al.^76^, to distinguish between internal and external attention, observed an increase in right-parietal alpha during a task requiring inner attention, which might represent inhibition of the ventral attention network.

In the framework of inter-group analysis, we estimated the spectral Granger Causality to investigate the alpha-band connectivity of low vs high comfort perception. To the best of our knowledge, this is the first study that exploited Granger Causality to differentiate the brain circuits of comfort perception arising during the exploration of a virtual aircraft cabin.

Although limited by the analysis at the scalp level, the present results underline differences in alpha-band connectivity between the two groups G1 and G2, which involve connections among frontal, temporoparietal, and occipital nodes. These differences likely involve attention networks. More particularly, in *r1VR* subjects of group G2 (who perceived higher comfort) exhibit a posterior network, and more robust top-down connectivity driven by the left frontal and central areas (electrodes F3 and C3) towards the occipital regions. Conversely, subjects in group G1, which tend to have a more right-than-left frontocentral alpha power in phase r1VR (see Fig. 6), show greater connectivity from left regions towards electrodes in the right centrotemporal zone (electrodes C4 and T8), together with a pretty negligible top-down connectivity from the temporal to the occipital electrodes.

Differences in lateralization are reported in the literature between normal and psychopathological subjects or subjects with different personality traits. These differences can be a clinical or a trait index^77^. Alpha power lateralization, in turn, can be a consequence of weaker top-down regulation and more robust (and asymmetric) frontocentral regulation, which overall may underlie a state of hypervigilance and hyperarousal. Hence, although all participants in the experiment are neurotypical, it is possible that individuals in group G1 are characterized by personality traits more prone to stress and anxiety when subjected to unknown experiences, given higher left–right lateralization and weaker top-down regulation.

Another crucial aspect is that group G2 in *r2VR* maintained almost the same top-down regulation mechanism observed in *r1VR*, and the same posterior network, reflecting the stability already highlighted in the power analysis. Conversely, group G1 changes its network from a frontal circuit in *r1VR* to a posterior circuit in *r2VR*, approaching that of G2. Probably, the switch of group G1 network after the interaction with the virtual cabin (*r2VR*) is related to the alpha power increase that we observed especially in the FCT region in the same phase, where G1 became more similar to G2. The latter, in turn, can be an effect of previous sensorimotor activity, as discussed above.

In conclusion, the present study demonstrates that EEG alpha power changes induced by a VR environment can be employed to discriminate between subjects’ *visual* comfort perceptions (low vs. high), as emerging from the subjective questionnaire. Specifically, we showed that subjective comfort perception can be predicted with a high statistical significance by looking at alpha ERD changes in the occipital zone. Indeed, subjects who reported a lower comfort or an insufficient appreciation of the VR aircraft cabin were also characterized by a greater alpha power ERD during the VR-stimulation phases, suggesting that alpha power can be a sensitive objective metric for comfort-level stratification. This opens a new perspective for using alpha power as a tool to discriminate between different levels of stress/comfort.

Moreover, we performed a connectivity analysis through the Granger Causality estimator, highlighting how brain circuits differ between higher and lower visual comfort perception. Specifically, we showed that a robust top-down regulation is characteristic of the high comfort perception, while a more left-to-right mechanism is distinctive of the low comfort perception in a virtual aircraft cabin. These aspects can represent a novel result that can stimulate future studies and improve our understanding of the neurophysiological mechanisms and brain networks underlying visual comfort.

A possible hypothesis, which requires further analysis, is that when a higher comfort is experienced, top-down mechanisms modulate visual attention, reducing the awareness or sensitivity toward external stimuli and restoring a status with greater partial relaxation.

Of course, we cannot be sure whether the differences between G1 and G2 are determined only by a different appreciation of the specific cabin design (as reported in the questionnaires) or also by a different attentive response/awareness/stress, more generally arising from the VR setting and experience. Indeed, it is possible that subjects who were more stressed by the VR experience, characterized by higher arousal and attention level, also reported a lower cabin appreciation. However, this does not bias the results of our work, whose objective was to test the possibility of discriminating between the subjects’ visual perceived comfort using EEG alpha power and assess possible connectivity differences.

Overall, this study can contribute to understanding the role of the alpha brain rhythm in cognitive processes, as well as the mechanisms which drive its modulation. These notions may be of great relevance in the perspective of future studies oriented to a Human-centered design approach, which aims to quantitatively assess subjects' perception of comfort during their interaction with an environment.

## Supplementary Information

Supplementary Information.

## Data Availability

The data used to support the findings of this study are available (in anonymized form) upon request submitted to Giulia Ricci (giulia.ricci29@unibo.it) and Francesca De Crescenzio (francesca.decrescenzio@unibo.it).
