# Supplementary material for: Relationship between electroencephalographic data and comfort perception captured in a Virtual Reality design environment of an aircraft cabin
Source: Sci Rep. 2022 Jun 29;12:10938. doi: 10.1038/s41598-022-14747-0 (PMC9243066; doi:10.1038/s41598-022-14747-0)
Supplement: Supplementary file 1 — Supplementary Information. [file 41598_2022_14747_MOESM1_ESM.docx]

**Supplementary Materials:**

**Extended Method Version**

**Relationship between electroencephalographic data and comfort perception captured in a Virtual Reality design environment of an aircraft cabin**

**Giulia Ricci^1*^, Francesca De Crescenzio^2^, Sandhya Santhosh^2^, Elisa Magosso^1§^, Mauro Ursino^1§^**

**Participants**

The experiments took place at the VLab of the University of Bologna, located at the Department of Industrial Engineering at the Forlì campus. Thirty-one healthy volunteers (22 males, 9 females) were recruited from university students. The age of participants ranged from 18 to 33 (mean ± std= 22.3 ± 3.5). All participants were right-handed and had normal or corrected to normal vision and reported no medical or psychiatric illness. This study was approved by the local Bioethics Committee of the University of Bologna (file number: 187339, year: 2018) and performed in accordance with the relevant guidelines and regulations. Each participant signed an informed consent prior to the start of the experiment and all data were analysed and reported anonymously.

**VR Environment and simulation Scenario**

VR systems can be classified according to the level of immersion, the level of presence and the level of intrusiveness of the devices. Moreover, VR systems are divided in Head Mounted Displays and projection-based displays. The first are very popular for their portability, for the level of immersion, for high end graphic capabilities and for their affordability. These are diffused in many application fields, ranging from videogaming to training or storytelling. The second type of VR systems are widely diffused in large companies for collaborative design sessions. In this class, the CAVE is a partially immersive visualization system that allows a high level of presence of the user. It is based on multiple rear projected screens that replicate the walls and, in some case, also the ceiling and the pavement ^1^. It is an efficient tool for interactive design review sessions or for those experiments that aim to quantify not only the visual feedback but also the interaction feedback. For the experiments of CASTLE project, a CAVE Automatic Virtual Environment has been used.

The CAVE facility at the University of Bologna is a CAVE-like virtual environment designed to recreate a sense of immersion by means of three, rear-projected, flat screens. Each screen covers a 2.5 m x 1.5 m area. Therefore, the total projection area is 7.5x1.5 m2. Multi-display functionalities are activated through a NVIDIA® 3D Vision® Pro architecture while stereoscopy is implemented by active shutter glasses compatible with the NVIDIA systems.

The active stereoscopy is enabled through shutter glasses. To allow the cabin environment to be navigated from a first-person perspective by a user moving on the CAVE floor, face and body tracking is implemented by capturing and filtering data provided by a Microsoft Kinect sensor placed in front of the user at the bottom of the CAVE central screen. The CAVE can provide basic key elements of a VR experience such as: immersion, feedback, interactivity, and multiple user collaboration ^2^. CAVE systems allow the navigation of the virtual scene, with a high presence level (the user can experiment his own within the VR scene). Moreover, in the CAVE the user only needs to wear the stereo glasses. This means that other devices, such as the EEG cap, can be worn without interfering with VR Head Mounted Displays resulting in a very cumbersome device set.

The different CAD models produced in the framework of the CASTLE project are representative of the cabin items of interest such as the fuselage, the seat, the lavatory, the galley, the cabin lining, the Flight Attendant Seat and the stowage bins. The CAD files are processed in order to simplify the hierarchy and to assign the model decomposition level according to the VR visualization and interaction requirements. Finally, these are imported in the simulation platform. The position and movement of the user's joints and face are tracked by the Microsoft Kinect system and reproduced by an avatar displayed in the virtual environment.

Moreover, in the lower bottom corner of the right screen, an exocentric view of the avatar is reproduced in order to support the user’s proprioception. Finally, to simulate the collision with objects of the virtual environment, the system sends visual and audio feedback each time the avatar virtually touches some selected parts of the model.

Due to the long development cycle, different experimental campaigns have been conducted during the project. The campaigns can differ for updates in the models or in the CMF (Colour and Material Finishing) features provided by designers. In the experimental campaign held in autumn 2019, for the purpose of this work, the experimental protocol has been integrated with the acquisition of EEG data. Therefore, the scenario described above, has been experienced by each participant during the interaction phase of the experiment, as described in the following paragraph.

**Experimental Protocol**

Each participant underwent a single experimental session. During the entire session, the participants were equipped with a wireless EEG device and they were asked not to speak and to limit their movements, with the exception of one phase (the *interaction* phase) where they were explicitly asked to move and interact with the virtual cabin.

In order to avoid to distract the participants and enhance their sensory immersion, the lights of VR laboratory were kept off and operators stayed in the background throughout the entire session.

Before the start of the main experimental session, the baseline (*base*) EEG signal was acquired for each participant. It consisted of a 5-min resting-state with eyes open, during which the participants seated centrally to the CAVE at approximately two metres away from the screens.

During this phase, the participants were not subjected to any stimulation, neither visual nor auditory, as VR screens were kept off and no sound was played. This phase has been explicitly designed to elicit a baseline relaxation state in each participant.

At the end of baseline phase, the participants, after wearing the shutter glasses, were asked to stand on the CAVE platform and were briefly trained to navigate the virtual environment, in order to familiarize with the avatar and with the interactive features of the virtual cabin (auditory and visual feedbacks of collision effect). It should be noted that the virtual scenario used during this training phase (a visual stowage on a black background) was different from those of the main experimental session.

The subsequent main experimental session was structured into four phases, with a total duration of 18 minutes.

The first phase, named *r1*, consisted in a 5-min eyes-open resting state without VR stimulation. As in the baseline acquisition, the participants were seated centrally to the CAVE, with the difference that a background sound, simulating an aircraft in motion, was turned on and was kept on for the rest of the experimental session. The second 5-min phase, named *r1VR*, consisted in a first static VR immersion phase. Here, the VR screens were switched on and were kept on until the end of the session. During this phase, the participants remained seated while visually exploring the static VR environment in which they were immersed, trying to capture both spatial and aesthetic aspects (Fig. S1, left panel).


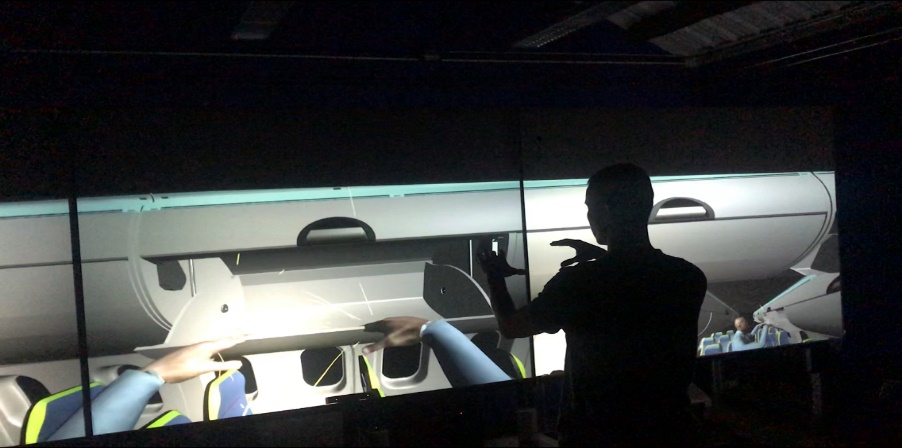


**Figure S1.** Left panel: participant sitting in the static VR environment (r1VR). Right panel: interaction of the subject with the virtual cabin

Specifically, the presented scenario consisted in an immersive view of a Regional Cabin Aircraft including a part of the aisle, the seats and the stowage bins. The third 3-min phase consisted in the *interaction* of the participants with the virtual cabin. Here, the participants stood up and moved in order to explore spaces and interact with the objects of the environment (Fig. S1, right panel). To this end, the chair was temporarily removed from the CAVE to allow a better navigation. Finally, the last 5-min phase, named *r2VR*, consisted in a second static VR immersion phase. During this phase, the experimental conditions were the same as in the first VR immersion phase (*r1VR*), with the participants seating in front of the screens and in the same static scenario shown previously.

At the end of the experiment, each participant filled in a questionnaire (see section 2.4) for subjective rating of comfort induced by the aesthetic and spatial features of the VR cabin shown during the main experimental session.

The EEG signals acquired in this experiment were used to investigate whether alpha-band EEG features (power and connectivity) during VR immersion differed depending on the subjective level of comfort (low vs high) elicited by the VR environment, as emerged from the questionnaire. It is worth noticing that the interaction phase (int) was excluded from the analyses. Indeed, in this phase, EEG signals were strongly corrupted by movement artifacts that couldn’t be corrected in a reliable way. Moreover, during this phase additional motor mechanisms were involved, influencing brain rhythms and likely confounding the effects related to comfort sensation. This does not mean that the effects of interaction phase were neglected: indeed, during the post-interaction phase (r2VR), comfort and appreciation of the VR environment, and related EEG features, were likely affected by the outcome of this interaction.

**Questionnaire**

The questionnaire consisted of 9 questions in total (Table S1). For the first 8 questions the subject had to answer the question “Please, express how much you agree with each statement” using a 5-point Likert scale, where 1=strongly disagree, 2=mostly disagree, 3=slightly agree, 4=mostly agree, 5=strongly agree. In these questions, participants were asked to evaluate the spatial and aesthetic properties of some cabin features, such as seats, cabin lining and stowage bin. The last question ('How much did you like the cabin?') had a wider scale, ranging from a minimum of 1 to a maximum of 10, and had the aim of capturing the participant's overall appreciation of the cabin. In this case, since the question was more general, a broader scale was chosen to allow subjects to define their level of appreciation more precisely.

| 1. The seat seemed easy to access | SEAT |
| --- | --- |
| 2. The seat appeared to have enough space to stretch my legs |  |
| 3. I had the feeling of being in a spacious environment |  |
| 4. The space to access the seat and the leg room appeared sufficient |  |
| 5. I was pleased with the style/aesthetics of the cabin lining | **CABIN**  **LINING** |
| 6. I was pleased with the style/aesthetics of the stowage bins | **STOWAGE BIN** |
| 7. The stowage bin appeared spacious enough to easily load my luggage |  |
| 8. The stowage bin seemed easy to reach/use |  |
| 9. How much did you like the cabin? | **OVERALL CABIN LIKING**  **(10 points)** |

**Table S1:** Overall cabin scenario questionnaire.

**EEG Acquisition and Pre-processing**

EEG signals were collected from all participants using a wireless EEG device that allowed the full mobility of participants in the VR laboratory. Specifically, we employed the OpenBCI Cyton board embedded with the OpenBCI Daisy Module (OpenBCI, https://openbci.com/) system, that register EEG signals by communicating wirelessly via the OpenBCI USB transmitter/receiver using RFduino radio module.

Fourteen wet Ag/AgCl electrodes (electrodes (F3, F4, C3, C4, T7, T8, Pz, PO3, PO4, PO7, POz, PO8, O1, and O2) embedded in an EEG cap were connected to the OpenBCI Cyton + Daisy Board device. The device was then fixed to the right arm of the participant. The electrodes were referenced to the right earlobe and grounded to the left earlobe. For all the participants, the fourteen EEG signals were online digitized at a sampling rate of 125 Hz and with a 24-bit resolution. Finally, signals were stored in a Matlab-compatible format. All EEG data were pre-processed offline using MATLAB R2020b (MathWorks Inc., Natick MA, USA). First, a 0.75-60 Hz band-pass filter was employed to retain the relevant EEG spectral content, and a 50 Hz notch filter was used to remove electric coupling interferences. Then, the 3-min portion of filtered signals corresponding to the *interaction* phase were removed and the remaining phases of the experiment, including the baseline signal, were concatenated (*base*, *r1*, *r1VR*, *r2VR*) along the time dimension. EEG artifact removal was performed via Independent Component Analysis (ICA), using the extended Infomax algorithm implemented in the open-source Matlab toolbox EEGLAB (https://sccn.ucsd.edu/eeglab/index.php). Then, artifactual components were visually identified and removed. At this stage, to avoid compromising the results of the study, one subject was excluded from further analysis due to the presence of a severe ECG artifact. In this case, the ICA algorithm failed in isolating the ECG artefactual component, preventing its removal from the relevant EEG signal. In addition, a second subject was excluded as it proved to be an outlier in subsequent steps, showing an extremely high variability in all experimental phases and risking to bias the sample trend. Overall, an average of 4.8 (SD = 1.2) ICs were rejected across a total of 29 participants. Subsequently, the remaining independent components were back-projected for the reconstruction of artifact-cleaned EEG signals. For each participant, the 5-min EEG data segments corresponding to the phases *base*, *r1*, *r1VR*, *r2VR* were extracted.

Moreover, to fine the precision of the frequency-domain analyses, the Individual Alpha Window (IAW) of each participant was identified. This choice was motivated by previous studies on interindividual differences in the alpha frequency band ^3–5^ showing that the standard alpha band (8-14 Hz) may not be appropriate for all individuals. For this purpose, the automated method of resting-state individual alpha-band quantification, recently proposed by Corcoran et al., ^6^ was applied to the *base* signals of each participant. Across the participants, we obtained f1 = 7.52 Hz ± 0.83 Hz (mean ± std, range = 5.7 Hz ÷ 9.5 Hz) and f2 = 14.0 Hz ± 0.99 Hz (mean ± std, range = 10.0 Hz ÷ 15.4 Hz). Then, for each participant and each phase the IAW was employed for alpha-band analyses.

**Alpha Power Computation**

For each participant and for each 5-min phase (*base, r1, r1VR, r2VR*) Power Spectrum Density (PSD) of each channel was computed by applying Welch’s periodogram method, by using a Hamming window of 5 seconds at 50% overlap, zeropadded to 10 seconds in order to obtain 0.1 Hz frequency resolution. Then, alpha power over the IAW was computed for each of the fourteen EEG electrodes and the values achieved were employed to realize scalp maps of alpha distribution. Furthermore, alpha power was also computed at a regional level. To this aim, channels were grouped into two macro regions of interest: the fronto-central-temporal region (FCT), obtained by aggregating the six antero-central electrodes (F3, F4, C3, C4, T7, T8), and the parieto-occipital region (OCC), obtained by aggregating the eight posterior channels (Pz, PO3, PO4, PO7, POz, PO8, O1, O2). Thus, an anterior and posterior Alpha Power was computed for each experimental condition. To stress the changes in alpha power compared with the resting state, for each phase the alpha power of each participant was normalized to the corresponding channel-level (for the *channel-wise* analysis) or regional-level (for the *regional-wise* analysis) value in the base period.

**Spectral Granger Causality analysis**

To further investigate the possible neural mechanisms of comfort perception induced by the VR environment, we evaluated the functional connectivity in the alpha band among the fourteen electrodes by using the frequency-dependent Granger Causality estimator, which provides directional metrics of functional connectivity. The Granger Causality estimator is based on the autoregressive (AR) modeling framework: considering two time series $x_{k,i}[n]$ and $x_{k,j}[n]$ (where *n* represents the discrete time) representing the activity at two distinct EEG electrodes ($E_{i}$ and $E_{j}$) for participant$k$, the Granger Causality estimator quantifies the causal interaction from $E_{i}$ to $E_{j}$ as the improvement in predictability of $x_{k,j}[n]$ when using a bivariate AR model (based on both past values of $x_{k,j}$ and past values of $x_{k,i}$) compared to a univariate AR model (based only on past values of $x_{k,j}$). Moreover, the frequency-domain Granger Causality is based on the spectral derivation of the bivariate process via the Fourier Transformation ^7^. The power spectrum of each time series $x_{k,j}[n]$ can be decomposed into an ‘intrinsic’ and a ‘causal’ part, considering the latter predicted by the other time series $x_{k,i}[n]$.

At each frequency $f$, the GC spectrum from $i$ to $j$ ${(GC}_{i\to j}(f))$ is defined as the logarithm of the ratio between the total power spectrum of $x_{k,j}[n]$ and the difference between the total power spectrum and the causal power predicted by $x_{k,i}[n]$. Therefore, the quantity ${GC}_{i\to j}\left( f \right)$ at a given frequency *f* is zero when the causal power of $x_{k,i}[n]$ onto $x_{k,j}[n]$ at *f* is zero and increases (>0) as the causal power increases.

For each participant and for each experimental condition, GC power spectra were computed between all pairs of electrodes and in both directions. In order to obtain a single GC value for each connection, the mean value of each spectrum was evaluated in the alpha band (considering the IAW) and these values were normalized to the corresponding values in *base* condition. Therefore, we tried to capture how the subject’s comfort/discomfort perception induced by aesthetic and spatial features of a virtual aircraft modulates the connectivity between EEG signals in the alpha band.

**Total Subjective Score and dichotomous subdivision**

Analysis on subjective data was carried out considering the scores of the questionnaire that each participant filled at the end of the experimental session. Hence, the nine scores (one for each question) were summed up for each participant, obtaining a *Total Subjective Score* (TSS). This value ranged from a minimum TSS value of 9, as the minimum assignable value to each question was 1, to a maximum TSS value of 50, as the maximum assignable value to eight out of nine questions was 5 and only for the last question was 10. In order to investigate differences in neural mechanisms underlying a different level of VR-induced comfort, subjects were divided in two classes by considering the median TSS value (*md*TSS=35): group G1 consists of participants (16) with a TSS less than or equal the median, i.e. who perceived lower comfort in the virtual cabin; group G2 consists of participants (13) with a TSS above the median value, i.e., who showed a greater appreciation of the virtual cabin.

**Statistical Comparison: Permutation Test**

The analysis of alpha power is presented in two steps.

For the first step of the analysis, we considered the entire set of participants (without group subdivision) and provided an overall assessment of how sensory stimulation in the different experiment phases affected alpha brain rhythm. For this purpose, we statistically compared the normalized alpha power in each of the three experimental conditions of background acoustic stimulation (*r1*), pre-interaction VR-immersion (*r1VR*) and post-interaction VR-immersion (*r2VR*) with the baseline condition (base), separately for each macro-scalp region (FCT and OCC). The statistical comparison of each phase vs baseline was performed by employing the two-tailed permutation-based t-test for dependent samples. It should be noted that in this case the two-tailed test was used as we had no a priori hypothesis on how the alpha power was varying (increase/decrease) compared to the baseline. To correct p-values for multiple comparisons, Bonferroni correction was applied (separately for each scalp macro-region).

The second step of the analyses was aimed to investigate whether different levels of comfort (lower and higher) elicited by the characteristics of the VR cabin were associated with differences in alpha-band EEG features (power and connectivity) during VR immersion. Since r1 did not involve VR immersion, these analyses were performed on phases r1VR and r2VR, by statistically evaluating differences between the two groups of participants (G1 and G2) in each phase. Specifically, the normalised alpha power, both *regional-wise* and *channel-wise,* was statistically compared by using the one-tailed permutation-based t-test for independent sample. In this context, the choice of the one-tailed test was made, as we hypothesised that subjects who particularly enjoyed the cabin (G2) also felt more relaxed during its visual exploration, thus showing greater alpha-band power. In case of *regional-wise* analysis, Bonferroni correction was adopted, separately for each scalp region. Instead, for *channel-wise* comparison (14 comparisons, one per channel), p-value correction was performed based on the maximum t-statistic, considering each phase separately. Finally, the statistical comparison of the normalised alpha-band GC between the two groups was performed using the one-tailed non-parametric permutation test for independent samples. In this case, uncorrected p-values were considered, due to the high number of involved variables, i.e. 14x13 connections, making correction requirement highly demanding; however, different levels of significance were distinguished (see section 3.3).

**References**

1. Cruz-Neira, C., Sandin, D. J. & DeFanti, T. A. Surround-screen projection-based virtual reality: the design and implementation of the CAVE. in *Proceedings of the 20th annual conference on Computer graphics and interactive techniques* 135–142 (1993).

2. Muhanna, M. A. Virtual reality and the CAVE: Taxonomy, interaction challenges and research directions. *Journal of King Saud University-Computer and Information Sciences* **27**, 344–361 (2015).

3. Haegens, S., Cousijn, H., Wallis, G., Harrison, P. J. & Nobre, A. C. Inter-and intra-individual variability in alpha peak frequency. *Neuroimage* **92**, 46–55 (2014).

4. Cecere, R., Rees, G. & Romei, V. Individual differences in alpha frequency drive crossmodal illusory perception. *Current Biology* **25**, 231–235 (2015).

5. Chiang, A. K. I., Rennie, C. J., Robinson, P. A., Van Albada, S. J. & Kerr, C. C. Age trends and sex differences of alpha rhythms including split alpha peaks. *Clinical Neurophysiology* **122**, 1505–1517 (2011).

6. Corcoran, A. W., Alday, P. M., Schlesewsky, M. & Bornkessel-Schlesewsky, I. Toward a reliable, automated method of individual alpha frequency (IAF) quantification. *Psychophysiology* **55**, e13064 (2018).

7. Chicharro, D. On the spectral formulation of Granger causality. *Biological cybernetics* **105**, 331–347 (2011).
